# Dissemination and implementation of evidence-based programs for people with chronic disease: the impact of the COVID-19 pandemic

**DOI:** 10.3389/fpubh.2023.1276387

**Published:** 2024-01-11

**Authors:** Peter Coyle, Jennifer Tripken, Subashan Perera, Gardenia A. Juarez, Lesha Spencer-Brown, Kathleen Cameron, Jennifer S. Brach

**Affiliations:** ^1^Department of Physical Therapy, University of Pittsburgh, Pittsburgh, PA, United States; ^2^National Council on Aging Center for Healthy Aging, Arlington, VA, United States; ^3^Department of Medicine and Biostatistics, University of Pittsburgh, Pittsburgh, PA, United States; ^4^Administration for Community Living, Washington, DC, United States

**Keywords:** implementation, chronic disease management, community programs, COVID-19, aging

## Abstract

**Background:**

Using the RE-AIM (Reach, Effectiveness, Adoption, Implementation, and Maintenance) framework, we describe the implementation of evidence-based chronic disease self-management education (CDSME) programs by the Administration for Community Living CDSME Grantees during 2016–2022 and we also explore the impact of the COVID-19 pandemic on implementation.

**Methods:**

Grantees collected data before and after the implementation of the CDSME programs and contributed to the national data repository. Data components included workshop information, participant information, and organizational data.

**Results:**

The cohort consisted of 175,973 individuals who participated in 34 CDSME programs across 45 states. Participants had a mean ± SD age of 66.1 ± 14.8 years, were primarily female (65.9%) and had a mean ± SD of 2.6 ± 2.3 chronic conditions. Compared to the pre-COVID-19 strata, those who participated during COVID-19 were on average 1.5 years younger and had slightly less comorbidities. For individuals who had pre and post program self-reported health, 65.3% stayed the same, 24.4% improved, and 10.3% worsened (*p* < 0.001) after participating in CDSME programs.

**Conclusion:**

CDSME offers a variety of programs across a broad geographic area to a diverse set of older adults in the US, underscoring the expansive reach of this public health initiative. COVID-19 appears to have shifted participant reach toward a slightly younger and healthier population. Finally, these programs appear to be effective in improving participants’ self-rated health. However, these results should be interpreted with caution, given limitations due to missing data and the observational nature of this study design.

## Introduction

Chronic disease is a common and costly problem in the United States with more than half of the US population having at least one chronic condition, and up to one-third having more than one (1, 2). The prevalence of chronic disease increases with age and in those 60 years of age or older, virtually everyone has at least one chronic disease and 80% have two or more (i.e., multimorbidity) (3). The health consequences are dire, as chronic diseases often lead to a loss of functional independence, (4–6) social isolation, (7) cognitive decline, (3, 8) and mortality (5, 6). In addition, the cost associated with chronic disease is enormous (9), with more than two-thirds of all healthcare costs go toward treating patients with chronic illnesses, and 95% of all Medicare spending is associated with chronic conditions (10).

Optimal chronic disease management has become the holy grail for patients, providers, policymakers, population health experts, and the research community (10, 11). Though each chronic disease provides a unique experience, many overlap in the behavioral risk factors (i.e., physical inactivity, poor nutrition, tobacco-use, and excessive alcohol consumption) that contribute to the development and progression of these conditions. Evidence-based chronic disease self-management education (CDSME) programs are public health interventions that target known behavioral risk factors by promoting behavior change within community networks. Evidence-based CDSME programs differ in their specific focus and may include various elements, including exercise and skills training. These efficacious programs have been rigorously studied and translated into practice models for community-based implementation (12). Analyses of specific CDSME programs have also demonstrated economic benefits (13–16). For example, participation in Stanford Chronic Disease Self-Management Program™ resulted in net benefit of $364 per participant, which translates to $8.9 billion if just 10% of the population with chronic disease participated (13).

Since the early 2000s, the Administration on Aging (AoA) has been building a comprehensive infrastructure for the delivery and sustainability of various evidence-based health promotion programs to support healthy aging. Since 2012, the Administration for Community Living (ACL), an operating division of the U.S. Department of Health and Human Services, has received dedicated funding through the Affordable Care Act’s Prevention and Public Health Fund to support dissemination of evidence-based CDSME programs. The ACL awards competitive grants in the form of cooperative agreements to states, community-based organizations, tribal entities, and institutions of higher education to support capacity building, scaling, and sustainability of evidence-based CDSME programs. Since 2017, the ACL has awarded approximately $38 million in cooperative agreements to support CDSME program dissemination and implementation.

While specific evidence-based CDSME programs have been studied in detail, implementation has generally been evaluated on an individual program or single organization basis. Much can be learned about the collective implementation of evidence-based CDSME programs over time. Implementation evaluation can provide an overview of real-world success and identify targets to guide future efforts for communities and policymakers. Furthermore, evaluation of recent data can help illustrate the impact of the COVID-19 pandemic on the implementation of these programs. Early in the pandemic, community programming was suspended, but as the pandemic progressed the use of remote delivery options, such as video-conferencing, phone and mail, were implemented for some but not all programs (17). The return to in-person program delivery varied greatly by state and type of site. Documenting the impact of the pandemic on the reach of these programs may inform efforts to develop alternative delivery options that are acceptable and available to all individuals.

Using the Reach, Effectiveness, Adoption, Implementation, and Maintenance (RE-AIM) framework, we examine the implementation of the ACL-approved evidence-based CDSME programs. The RE-AIM framework is a commonly accepted model to plan and examine implementation success for various health promotion and healthcare initiatives across clinical, community, and work settings (18). Our specific purpose for these secondary analyses was to describe the reach and explore the effectiveness of these programs over recent years. We also explored how the COVID-19 pandemic, which had a well-known impact on communities (e.g., restrictions on in-person gatherings), affected reach and effectiveness.

Findings from this work will inform the efforts of the national CDSME network of partners. National CDSME Resource Center data can be used to examine the number, proportion, and representativeness of people who participated in an evidence-based CDSME program that was supported by the ACL. Furthermore, while these programs have been rigorously studied to ensure there are benefits to participants, evaluating these data allows for quality assurance (e.g., correcting program drift). Ultimately, these data are also crucial in the justification of ongoing support from policymakers and their constituents, which ensures the continuation of necessary funds to support this critical public health endeavor.

## Materials and methods

### Study participants and procedures

These analyses used data from the National CDSME Resource Center’s data repository. All ACL grantees are required to use this national database as a condition of funding. These data reflect various program workshop, delivery site, and participant information. Program workshop leaders collected these data, and organizations that hosted the workshops coordinated data entry into the repository in a centralized or decentralized manner at the state, regional or local level. Grantees employed methods of data collection, including paper-based, interviewer-administered, and electronic capture, that worked best for their individual location. All participant-specific data were de-identified and aggregated. For this study, data were limited to participants who started and completed a CDSME program in either January 1, 2016–December 31, 2019, or January 1, 2021 – October 4, 2022 (i.e., the last day the repository was updated before starting these analyses). To form clear subgroups that represented pre- and post- COVID-19 epidemic onset, data from participants who started or finished within the 2020 timeframe were excluded, because the degree and timing of specific public health restrictions varied by region. Additionally, it is important to note that these data reflect programs implemented by ACL CDSME grantees and, as such, do not include other areas or organizations that offered CDSME programs, not required to use the repository. This study which involved secondary data analysis was deemed exempt by the University of Pittsburgh Institutional Review Board as it did not include identifiable data.

### Data and measures

#### Program characteristics

The national data repository includes more than 30 different evidence-based CDSME programs. The programs included in this analysis were those that were deployed by ACL grantees during the timeframe of interest (i.e., 2016–2019 or 2021–2022).

#### Delivery site characteristics

The organizational infrastructure where CDSME programs were offered was diverse, and grantees classified the delivery sites as one of the following setting types: senior center; residential facility; healthcare organization; faith-based organization; community center; parks and recreation/other recreational organization; Area Agency on Aging/multi-purpose social services/state unit on aging; library; county health department/municipal government/state health department; educational institution; tribal center; workplace; other. The state and county where each workshop was implemented was also reported. Most grantees offered more than one program and reached multiple implementation sites.

#### Participant characteristics and outcomes

Grantees collected participant information during an orientation session (if applicable) or the first program session, as well as upon program completion. Though encouraged by workshop leaders, individuals were not required to complete the participant survey to participate in the program. During the pre-program survey (i.e., baseline), participants reported their age, sex, race/ethnicity, education level, and living arrangements. Participants also reported the presence/absence of 16 different chronic conditions (e.g., heart disease, cancer), and whether they felt their activities were limited in any way due to physical, mental, or emotional problems.

Uniform outcome assessment is an evolving target for the National CDSME Resource Center, especially among CDSME programs, which have diverse structures and foci. Currently, participants are asked to answer the following questions (1) “in general, would you say that your health is excellent, very good, good, fair or poor?” (i.e., self-rated health) and (2) “how often do you feel lonely or isolated from those around? Always, often, sometimes, rarely, or never?.” Self-rated health was introduced to the pre- and post-program surveys in November 2016 and August 2017, respectively. Frequency of feeling lonely or isolated was introduced to both pre- and post-program surveys in February 2020. For these analyses, we considered self-rated health to be our primary exploratory outcome, given the longer history of its use in CDSME outcome assessment and its robust prognostic properties regarding future health events. We considered frequency of feeling lonely or isolated to be our secondary exploratory outcome.

### Statistical analyses

Statistical analyses were performed using SAS version 9.4 (SAS Institute, Inc., Cary, NC). Descriptive analyses included means and standard deviations for continuous variables, as well as frequencies and proportions for categorical variables. Independent samples *t* test and chi-squared tests were used to compare those who participated pre-COVID (i.e., 2016–2019) and during COVID (i.e., 2021–2022) on all pre-program characteristics. To explore overall effectiveness of CDSME programs, we restricted our analytic cohort to those who completed pre- and post-program self-rated health, and we compared them to those who only completed pre-program self-rated health to assess any dropout bias. Among this restricted analytic cohort, we then used Bowker’s symmetry tests to compare pre- vs. post-program self-rated health and frequency of feeling lonely or isolated.

## Results

### Reach

#### Program level

The number of participants by CDSME program is displayed in Table 1. The cohort consisted of 175,973 participants who participated in 34 CDSME programs. The programs with the most participation included the Chronic Disease Self-Management Program (39.6%), the Diabetes Self-Management Program (28.2%), and the Chronic Pain Self-Management Program (8.9%). We also examined how program participation changed from pre-COVID to during COVID. Descriptively, the proportion of individuals who participated in those three programs declined from pre-COVID to during COVID timeframes, while the proportion of individuals who participated in HomeMeds and Walk with Ease increased.

**Table 1:** Participants by program.

Program	Total cohort (*n* = 175,973)	Pre-COVID (2016–2019) (*n* = 152,891)	During COVID (2021–2022) (*n* = 23,082)
	*n* (%)		
Active living every day	43 (<0.1)	43 (<0.3)	0 (0)
Arthritis foundation aquatic program	16 (<0.1)	0 (0)	16 (0.1)
Arthritis foundation exercise program	1,014 (0.6)	115 (0.1)	899 (3.9)
Arthritis self-management program	77 (<0.1)	77 (0.1)	0 (0)
Better choices, better health	834 (0.5)	474 (0.3)	380 (1.6)
Camine Con Gusto	50 (<0.1)	50 (<0.1)	0 (0)
Cancer: thriving and surviving	1,736 (1.0)	1,511 (1.0)	225 (1.0)
Chronic disease self-management program	69,672 (39.6)	65,295 (42.7)	4,377 (19.0)
Chronic pain self-management program	15,593 (8.9)	13,385 (8.8)	2,208 (9.6)
Diabetes self-management program	49,659 (28.2)	45,776 (29.9)	3,883 (16.8)
EnhanceFitness	138 (0.1)	138 (0.1)	0 (0)
EnhanceWellness	5 (<0.1)	5 (<0.1)	0 (0)
Fit and strong!	344 (0.2)	282 (0.2)	62 (0.3)
Health coaches for hypertension control	1 (0)	0 (0)	1 (<0.1)
Healthy IDEAS	473 (0.3)	418 (0.3)	55 (0.2)
HomeMeds	11,001 (6.3)	7,119 (4.7)	3,882 (16.8)
Mind over matter	429 (0.2)	194 (0.1)	235 (1.0)
PEARLS	134 (0.1)	0 (0)	134 (0.6)
Positive self-management program	116 (0.1)	116 (0.1)	0 (0)
Powerful tools for caregivers	1,531 (0.9)	1,445 (1.0)	86 (0.4)
Programa de Manejo Personal de Dolor	24 (<0.1)	0 (0)	24 (0.1)
Programa de Manejo Personal de la Artritis	12 (<0.1)	12 (<0.1)	0 (0)
Programa de Manejo Personal de la Diabetes	5,347 (3.0)	4,686 (3.1)	661 (2.9)
Screening, Brief Intervention, and Referral to Treatment	495 (0.3)	252 (0.2)	243 (1.1)
Tomando Control de su Salud	6,991 (4.0)	6,215 (4.1)	776 (3.4)
Active living with chronic pain	99 (0.1)	0 (0)	99 (0.4)
Active living with diabetes	715 (0.4)	0 (0)	715 (3.1)
Active living with chronic conditions	2,135 (1.2)	455 (0.3)	1,680 (7.3)
Walk with ease	6,637 (3.8)	4,470 (2.9)	2,167 (9.4)
Wellness recovery action plan	254 (0.1)	171 (0.1)	83 (0.4)
Workplace chronic disease self-management program	388 (0.2)	177 (0.1)	211 (0.9)
Missing	10 (<0.1)	10 (<0.1)	0 (0)

#### Delivery site level

**Figure 1:**
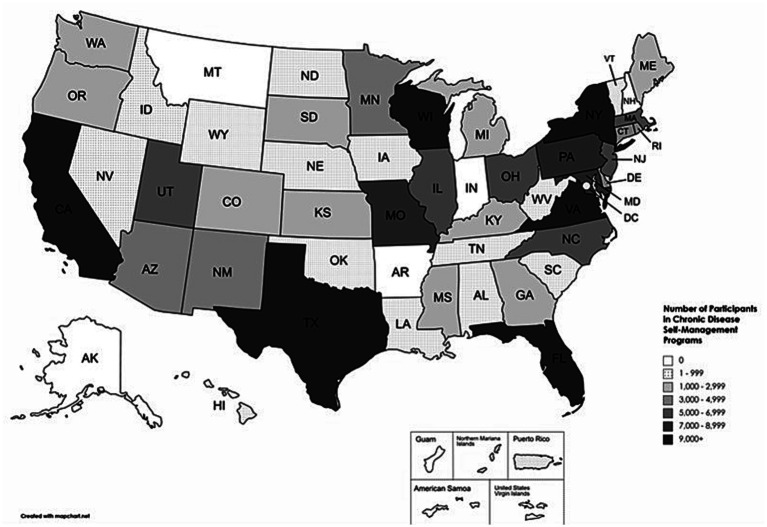
The number of individuals who participated in chronic disease self-management education programs by state.

The number of individuals who participated in CDSME programs by state is illustrated in Figure 1. States with the greatest number of participants (>9,000) were Florida, New York, Virginia, California, Wisconsin, and Texas. Table 2 displays the number of individuals who participated by setting type, with the most common settings being healthcare organizations (20.5%), senior centers (17.0%), and residential facilities (13.7%).

**Table 2:** Site level characteristics for all participants.

Setting	*N* (%)
Senior center	29,898 (17.0)
Residential facility	24,107 (13.7)
Healthcare organization	36,077 (20.5)
Faith-based organization	10,548 (6.0)
Community center	10,804 (6.1)
Parks and recreation/other recreational organization	5,336 (3.0)
Area agency on aging / multi-purpose social services / state unit on aging	11,118 (6.3)
Library	5,438 (3.1)
County health department / municipal government / state health department	4,786 (2.7)
Educational institution	10,599 (6.0)
Tribal center	578 (0.3)
Workplace	1,661 (0.9)
Other	23,392 (13.3)
Missing	1,631 (0.9)

#### Participant level

Participant characteristics for the total cohort (*n* = 175,973), pre-COVID strata (i.e., 2016–2019; *n* = 152,891), and during COVID strata (i.e., 2021–2022; *n* = 23,082) are displayed in Table 3. Participants had a mean ± SD age of 66.1 ± 14.8 years and were primarily female (65.9%). Individuals from various racial and ethnic groups participated, but the cohort was primarily White (55.1%) and not Hispanic or Latino (69.4%). Nearly half of the participants reported attending some college or vocational school, and approximately 10% of the sample did not complete high school. Participants had a mean ± SD of 2.6 ± 2.3 chronic conditions of the 16 assessed. The most commonly reported chronic conditions were hypertension (40.4%), arthritis (34.2%), and diabetes (33.7%). Nearly one quarter of the cohort reported being limited in activities because of physical, mental, or emotional problems. Approximately one third of the cohort rated their general overall health as good, very good, or excellent.

**Table 3:** Pre-program characteristics of the total sample, those who participated from pre-COVID pandemic (i.e., 2016–2019), and those who participated during the COVID pandemic (i.e., 2021–2022).

Baseline characteristic	Total cohort (*n* = 175,973)	Pre-COVID (2016–2019) (*n* = 152,891)	During COVID (2021–2022) (*n* = 23,082)	
	Mean ± SD or *n* (%)			p*
Age, *y*	66.1 ± 14.8	66.3 ± 14.9	64.8 ± 14.5	<0.001
Sex				<0.001
Female	115,962 (65.9)	102,186 (66.8)	13,776 (59.7)	
Male	37,734 (21.4)	33,669 (22.0)	4,065 (17.6)	
Missing	22,277 (12.7)	17, 036 (11.1)	5,241 (22.7)	
Lives alone				<0.001
Yes	40,800 (23.2)	35,895 (23.5)	4,905 (21.3)	
No	65,168 (37.0)	56,696 (37.1)	8,472 (36.7)	
Missing	70,005 (39.8)	60,300 (39.4)	9,705 (42.1)	
Ethnicity				<0.001
Hispanic or Latino	20,521 (11.7)	18,003 (11.8)	2,518 (10.9)	
Not Hispanic or Latino	122,051 (69.4)	110,103 (72.0)	11,948 (51.8)	
Missing	33,401 (19.0)	24,785 (16.2)	8,616 (37.3)	
Race				<0.001
American Indian or Alaska Native	2,587 (1.5)	2,370 (1.6)	217 (0.9)	
Asian	5,588 (3.2)	4,810 (3.2)	778 (3.4)	
Black	29,976 (17.0)	27,156 (17.8)	2,820 (12.2)	
Native Hawaiian	712 (0.4)	672 (0.4)	40 (0.2)	
White	96,970 (55.1)	86,724 (65.7)	10,246 (44.4)	
Multiracial	2,272 (1.29)	2,084 (1.36)	188 (0.8)	
Missing	37,868 (21.5)	29,075 (19.0)	8,793 (38.1)	
Education				<0.001
≥ College graduate	33,632 (19.1)	34,779 (22.7)	4,875 (21.1)	
Some college or vocational school	44,096 (25.1)	39,819 (26.0)	4,277 (18.5)	
High school graduate or GED	36,654 (20.8)	33,790 (22.1)	2,864 (12.4)	
≤ Some high school	17,204 (9.8)	15,746 (10.3)	1,458 (6.3)	
Missing	44,387 (25.2)	34,799 (22.7)	9,608 (41.6)	
Chronic conditions (total)	2.6 ± 2.3	2.7 ± 2.2	2.1 ± 2.3	<0.001
Limited in any way in any activities because of physical, mental, or emotional problems				<0.001
No	75,034 (42.6)	74,270 (48.6)	764 (3.3)	
Yes	39,712 (22.6)	39,319 (25.7)	393 (1.7)	
Missing	61,227 (34.8)	39,302 (25.7)	21,925 (95.0)	

SD, standard deviation.

*Value of *p* for pre-COVID vs. during COVID comparisons.

Several statistically significant differences existed between the pre-COVID and during COVID strata, but in many cases, statistical significance is likely due to the large sample size rather than material between-group differences. However, some differences do bear mentioning. Compared to the pre-COVID strata, those who participated during COVID were slightly younger (64.8 versus 66.3 years) and had slightly less comorbidities (2.1 versus 2.7) on average. Furthermore, we should also note that missing data were common among most factors, and missingness tended to be greater during COVID relative to pre-COVID.

### Effectiveness

Table 4 illustrates the characteristics of the restricted cohort for exploring the effectiveness of CDSME programs. Of the 88,595 participants who completed pre-program self-rated health, 5,675 participants completed their post-program self-rated health (i.e., the restricted analytic cohort), while 82,920 participants did not. As noted above, post-program self-rated health was not introduced until August 2017, nearly 2 years after the start of the data collection. Thus, a certain degree of missingness for post-program self-rated health was to be expected by virtue of delayed deployment. Compared to those who only completed the pre-program self-rated health outcome, those who completed both pre- and post-program questions differed significantly on many characteristics. Like pre- and during COVID comparisons, however, many differences are likely due to large sample size rather than material between-group differences or differences in the degree of missingness (i.e., individuals who completed both pre- and post-program self-rated health tended to have more complete data). Of note, those who completed both pre- and post-program self-rated health were slightly younger than those who completed only pre-program self-rated health.

**Table 4:** Baseline characteristics for all participants who completed the preprogram self-rated health question, participants who completed both pre- and post-program self-rated health question, and those who only completed the pre-program self-rated health question (2016–2019, 2021–2022).

Baseline characteristic	Completed pre-program SRH question (*n* = 88,595)	Completed both pre- and post-program SRH question (*n* = 5,675)	Completed only pre-program SRH question (*n* = 82,920)	
	Mean ± SD or *n* (%)			p*
Age, *y*	66.0 ± 14.8	64.8 ± 14.5	66.1 ± 14.8	<0.001
Sex				<0.001
Female	65,554 (74.0)	4,579 (80.7)	60,965 (73.5)	
Male	20,584 (23.2)	1,204 (18.1)	19, 560 (23.6)	
Missing	2,467 (2.3)	72 (1.3)	2,395 (2.9)	
Lives alone				<0.001
Yes	30,251 (34.2)	2,018 (35.6)	28,233 (34.1)	
No	47,930 (54.1)	3,578 (63.1)	44,352 (53.5)	
Missing	10,414 (11.8)	79 (1.4)	10,335 (12.5)	
Ethnicity				<0.001
Hispanic or Latino	13,365 (15.1)	1,227 (21.6)	12,138 (14.6)	
Not Hispanic or Latino	67,622 (76.3)	4,039 (71.2)	63,583 (76.7)	
Missing	7,608 (8.6)	409 (7.2)	7,199 (8.7)	
Race				<0.001
American Indian or Alaska Native	1,601 (1.8)	66 (1.2)	1,535 (1.9)	
Asian	3,633 (4.1)	389 (6.9)	3,244 (3.9)	
Black	17,589 (19.9)	1,113 (19.6)	16,476 (19.9)	
Native Hawaiian	506 (0.6)	20 (0.4)	486 (0.6)	
White	55,754 (62.9)	3,667 (64.6)	52,087 (62.8)	
Multiracial	1,417 (1.6)	78 (1.4)	1,339 (1.6)	
Missing	8,095 (9.1)	342 (6.0)	7,753 (9.4)	
Education				<0.001
≥ College graduate	22,143 (25.0)	1,785 (31.5)	41,454 (50.0)	
Some college or vocational school	28,241 (31.9)	1,701 (30.0)	26,540 (32.0)	
High school graduate or GED	22,235 (25.1)	1,139 (20.1)	21,096 (25.4)	
≤ Some high school	11,028 (12.4)	844 (14.9)	10,184 (12.3)	
Missing	4,948 (5.6)	206 (3.6)	4,742 (5.7)	
Chronic conditions (total)	3.2 ± 2 0.2	3.2 ± 2.3	3.2 ± 2.2	0.900
Arthritis	36,317 (41.0)	2,278 (40.1)	34,039 (41.1)	0.178
Respiratory	15,280 (17.3)	973 (17.1)	14,307 (17.3)	0.834
Cancer	11,706 (13.2)	707 (12.5)	10,999 (13.3)	0.083
Chronic pain	22,829 (25.8)	1,777 (31.3)	21,052 (25.4)	<0.001
Depression	17,633 (19.9)	100 (1.8)	17,533 (21.1)	<0.001
Diabetes	36,185 (41.0)	2,203 (38.8)	33,982 (41.0)	0.001
Heart disease	13,532 (15.3)	742 (13.1)	12,790 (15.4)	<0.001
High cholesterol	35,686 (40.2)	2,452 (43.2)	33,234 (40.1)	<0.001
Hypertension	42,305 (47.8)	2,819 (49.7)	39,486 (47.6)	0.003
Kidney disease	4,162 (4.7)	356 (6.3)	3,806 (4.6)	<0.001
Multiple sclerosis	99 (0.1)	0 (0.0)	99 (0.1)	0.003
Obesity	16,534 (18.7)	1,631 (28.7)	14,903 (18.0)	<0.001
Schizophrenia/psychotic disorder	1,566 (1.8)	143 (2.5)	1,423 (1.7)	<0.001
Stroke	4,777 (5.4)	245 (4.3)	4,532 (5.5)	<0.001
Osteoporosis	12,059 (13.6)	868 (15.3)	11,191 (13.5)	<0.001
Other	13,403 (15.1)	962 (17.0)	12,441 (15.0)	<0.001
None	8,468 (9.6)	678 (12.0)	7,790 (9.4)	<0.001
Limited in any way in any activities because of physical, mental, or emotional problems				<0.001
No	49,089 (55.6)	462 (8.1)	48,627 (58.6)	
Yes	22,125 (25.0)	124 (2.2)	22,001 (26.5)	
Missing	17,381 (19.6)	5,089 (89.7)	12,327 (14.9)	
Self-Rated Health**				<0.001
Excellent	3,509 (4.0)	188 (3.3)	3,321 (4.0)	
Very good	17,269 (19.5)	983 (17.3)	16,286 (19.6)	
Good	40,237 (45.4)	2,585 (45.6)	37,652 (45.4)	
Fair	23,491 (26.5)	1,633 (28.8)	21,858 (26.4)	
Poor	4,090 (4.6)	286 (5.0)	3,804 (4.6)	
Frequency of feeling lonely or isolated***				<0.001
Always	209 (0.2)	108 (1.9)	101 (0.1)	
Often	741 (0.8)	380 (6.7)	361 (0.4)	
Sometimes	2,731 (3.1)	1,398 (24.6)	1,333 (1.6)	
Rarely	2,841 (3.2)	1,428 (25.2)	1,413 (1.7)	
Never	2,927 (3.3)	1,383 (24.4)	1,544 (1.9)	
Missing	79,146 (89.3)	978 (17.2)	78,168 (94.3)	

SRH, Self-Rated Health; SD, Standard Deviation.

*Value of *p* for completed both pre- and post-program self-rated health question vs. completed only pre.

**Added to the pre-program survey in November 2016 and post-program survey in August 2017.

***Added to both pre- and post-program surveys in February 2020.

**Figure 2:**
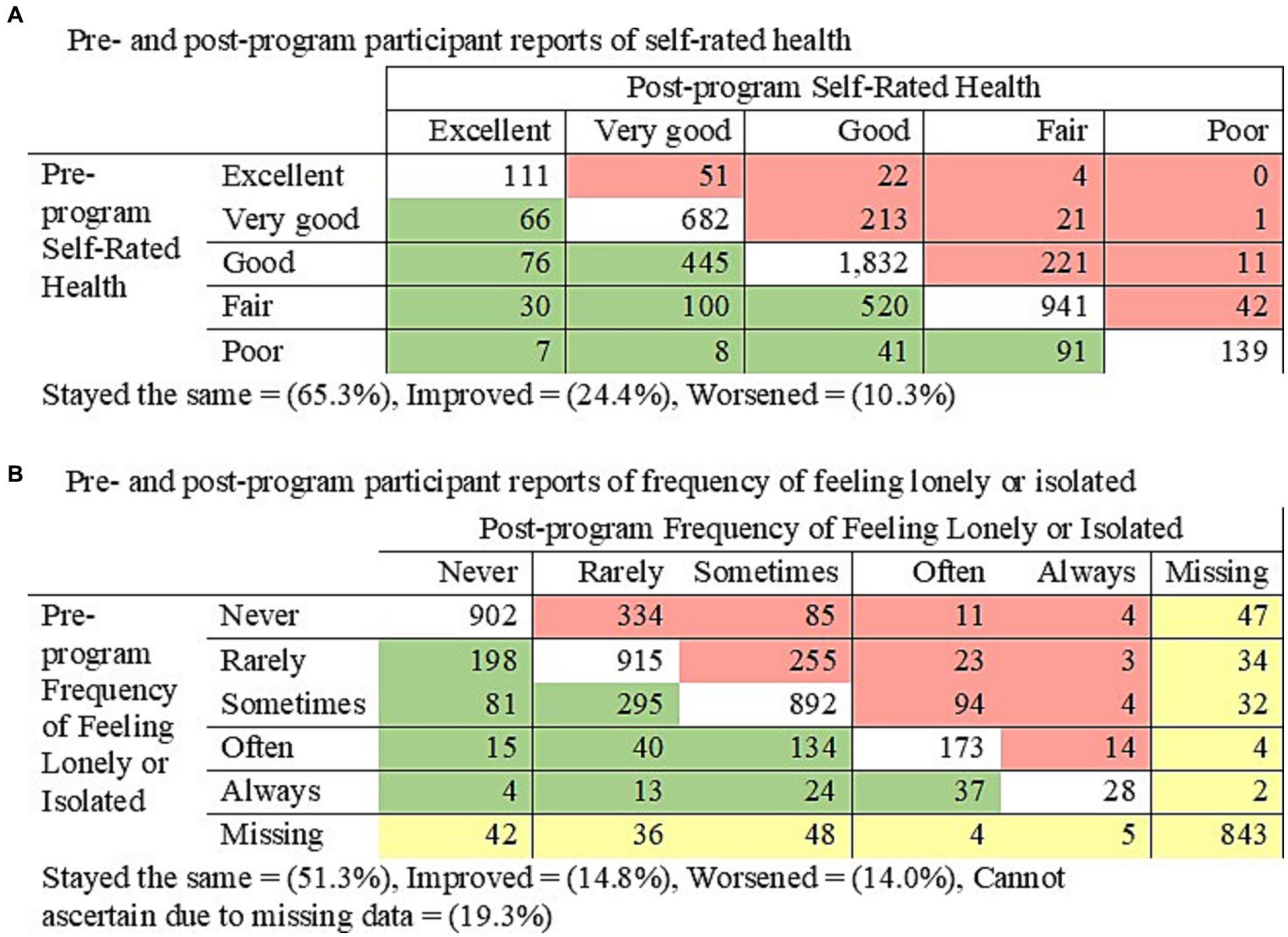
Symmetry tests for pre- to post-program **(A)** self-rated health (*p* < 0.001) and **(B)** frequency of feeling lonely or isolated (*p* < 0.001).

Figure 2 displays participants’ pre- and post-program self-rated health and frequency of feeling lonely or isolated for the analytic cohort. Cells highlighted in green indicate participants who improved from pre- to post-program, the white cells indicate those who stayed the same, and the red cells indicate those who worsened. For self-rated health, 65.3% stayed the same, 24.4% improved, and 10.3% worsened (*p* < 0.001). For frequency of feeling lonely or isolated, 51.3% stayed the same, 14.8% improved, 14.6% worsened, and 19.3% were missing either pre- or post-program data (*p* < 0.001).

## Discussion

During the nearly 6 years of CDSME program participation observed, the CDSME evidence-based programs reached over 175,000 older adults and adults with disabilities in 45 states, in addition to Washington D.C. and Puerto Rico. The CDSME programs were offered in numerous settings, including senior centers, residential facilities, and healthcare organizations. Though missing data on demographic variables prevent us from drawing definitive comparisons to the broader US older adult population, the sample contains participants of varying racial, ethnic, educational, and health backgrounds.

This dataset also allowed us to examine the impact COVID-19 had on CDSME reach. Shifts in program popularity from pre-COVID-19 to during COVID-19 was observed. For example, there was a decline in the proportion of people who participated in the Chronic Disease Self-Management Program (pre-COVID-19 = 42.7% vs. during COVID-19 = 19.0%), while the proportion of people who participated in the HomeMeds program increased (pre-COVID-19 = 4.7% vs. during COVID-19 = 16.8%). These shifts are unsurprising given the nature of program format, wherein some programs (e.g., Chronic Disease Self-Management Program) were often delivered in-person to groups of participants and others (e.g., HomeMeds) were delivered one-on-one or virtual (12). It should be noted that the COVID-19 pandemic led to innovations in program formatting (e.g., shifting to virtual models) (17). However, creating uniform data collection methods often lags behind changes in program implementation, and data reflecting program format were not consistently reported.

Shifts in participant demographics were also observed pre-COVID-19 vs. during COVID-19. Statistically significant differences were common across several participant characteristics (e.g., sex, living situation, ethnicity, and race). However, large sample sizes offer great statistical power, and many of these differences may not be material in nature. Furthermore, missing data prevents us from drawing definitive conclusions about shifts in participant demographics, and missing data appears to be more common since the COVID-19 pandemic. One notable difference, however, occurred with respect to age and number of chronic conditions. Compared to pre-COVID-19, those who participated during COVID-19 appear to be slightly younger and healthier. Though some programs provided alternative delivery formats that allowed for social distancing others did not. Thus, it is possible that older and less healthy adults may avoid in-person participation due to the potential COVID-19 exposure. In addition, alternative delivery formats that require technology may be a barrier to some older adults who do not have access to technology or are less comfortable using technology (17).

Of the approximately 175,000 participants in our sample, only 5,675 completed both the pre- and post-program assessments of self-rated health, our primary outcome of CDSME effectiveness. As mentioned before, post-program self-rated health assessment was not introduced into the data collection process until August 2017, thus, some missing data are to be expected. Nevertheless, our data indicate that there were significantly more improvements (24.4%) in self-rated health following program participation, compared to declines (10.3%). These findings are notable given the robust predictive nature of self-rated health (19–21). For example, in one cohort study, self-rated health had similar predictive capability of 10-year mortality rates compared to a comprehensive measure of objective health, which included assessments of the presence of various diseases, ability to perform specific activities of daily living, and psychological wellbeing (21). Self-rated health is a subjective indicator of health status that reflects various domains of health and wellbeing (e.g., biological, mental, social, and functional), which allows a person to weigh the importance of each underlying factor according to their own individual and cultural beliefs relative to health and health behaviors. Thus, this seemingly simple outcome measure should be an essential aspect of program effectiveness evaluation.

An ACL-approved “evidence-based program,” must have demonstrated efficacy using either an experimental or quasi-experimental design (22). Thus, the CDSME programs included in these analyses were shown to be effective in previous research studies across a variety of health outcomes. However, uniform outcome assessment that applies to all CDSME programs is difficult to obtain. Each CDSME program has a unique disease focus, approach, and goals. Given its independence from any one disease or condition, self-rated health is an attractive tool for assessing the effectiveness of CDSME programs without focusing on any specific disease or condition. Future efforts should be dedicated to identifying a comprehensive but parsimonious collection of meaningful outcomes that apply to all CDSME programs, which supplement self-rated health.

Of note, we found the proportion of people who improved (14.8%) and declined (14.6%) relative to how often they felt lonely or isolated. Feelings of loneliness and isolation are a serious health concern linked to chronic disease, (23) and we felt it important to include this as a possible outcome relative to program effectiveness. Moreover, many CDSME programs include a group and/or in-person component, which may lead to an expansion of social networks and thereby combat feelings of loneliness or isolation. The lack of clear improvements in this important outcome may have occurred for a variety of reasons. First, this measure was introduced in CDSME program data collection process in February 2020; in our analyses, only those who participated during COVID (i.e., 2021–2022) had data on this outcome measure. Second, feelings of social isolation may be only partially related to chronic disease status, and thus the impact of CDSME programs may be too small as to be discernable or material.

We should also note the significant limitations to our study and findings. First and foremost, the degree of missing data limits the ability to generalize and draw definitive conclusions from our results. Thus, we considered some of our analyses (i.e., effectiveness evaluation) to be exploratory in nature. Internal tracking by the National CDSME Resource Center staff (data not shown) indicate that grantees had challenges with several aspects of the data collection process. For example, grantees expressed concerns over the inefficient means of data collection (i.e., paper surveys), the time required to enter data into the database, program leaders’ compliance with reporting data in a timely manner, and staff turnover. These issues identify clear opportunities for process improvement, which may include streamlining data collection through virtual data capture (e.g., providing tablets to complete surveys), incentivizing data collection for program leaders and participants, and developing internal reporting processes for monitoring quality and completeness of collection. Such improvements could lead to more robust analyses that help determine for whom specific programs are most effective (i.e., precision public health).

In addition to missing data, we should also note limitations concerning our study design and analytical approach. From the perspective of reach, we examined geographical distribution at the state-level to ascertain a broad definition of reach. However, there is opportunity to examine geography precisely at the county and zip-code level of program delivery sites. These analyses may offer a more nuanced understanding of whether these programs are reaching under-resourced areas, where there is potentially a more urgent need for public health interventions. Also, our study design was observational in nature, and therefore causality cannot be definitively determined when evaluating effectiveness. Future research may consider quasi-experimental designs, if possible, to strengthen study conclusions.

Despite these limitations, there are notable strengths to our study. To our knowledge, this dataset is the only one of its kind, offering a unique ability to answer our research questions. Despite missing data, our findings were also robust, particularly with respect to exploring CDSME program impact on self-rated health. In addition, this dataset is diverse from a sociodemographic perspective, containing data from thousands of participants from traditionally underrepresented backgrounds. Thus, the findings from this study may be more generalizable to populations not traditionally captured in conventional research studies.

In conclusion, CDSME offers a variety of programs across a broad geographic area to a diverse set of older adults in the US, underscoring the expansive reach of this public health initiative. COVID-19 appears to have shifted CDSME program offerings toward those with formats that limit group exposure, and to have shifted participant reach toward a slightly younger and healthier population. Finally, these programs appear to be effective in improving participants’ self-rated health.

## Data availability statement

The original contributions presented in the study are included in the article/supplementary material, further inquiries can be directed to the corresponding author.

## Ethics statement

The studies involving humans were approved by University of Pittsburgh Institutional Review Board. The studies were conducted in accordance with the local legislation and institutional requirements. Written informed consent for participation was not required from the participants or the participants’ legal guardians/next of kin in accordance with the national legislation and institutional requirements. This study which involved secondary data analysis was deemed exempt by the University of Pittsburgh Institutional Review Board as it did not contain identifiable data.

## Author contributions

PC: Conceptualization, Writing – original draft, Writing – review & editing. JT: Conceptualization, Writing – review & editing. SP: Conceptualization, Formal analysis, Methodology, Writing – review & editing. GJ: Writing – review & editing. LS-B: Writing – review & editing. KC: Conceptualization, Supervision, Writing – review & editing. JB: Conceptualization, Investigation, Methodology, Supervision, Writing – review & editing.
